# Supplementary material for: Exocytosis of the silicified cell wall of diatoms involves extensive membrane disintegration
Source: Nat Commun. 2023 Jan 30;14:480. doi: 10.1038/s41467-023-36112-z (PMC9886994; doi:10.1038/s41467-023-36112-z)
Supplement: Supplementary file 1 — Supplementary Information [file 41467_2023_36112_MOESM1_ESM.pdf]

Supplementary information

Exocytosis of the silicified cell wall of diatoms involves extensive membrane disintegration

Diede de Haan, Lior Aram, Hadas Peled-Zehavi, Yoseph Addadi, Oz Ben-Joseph, Ron Rotkopf, Nadav Elad, Katya Rechav and Assaf Gal

Supplementary figures

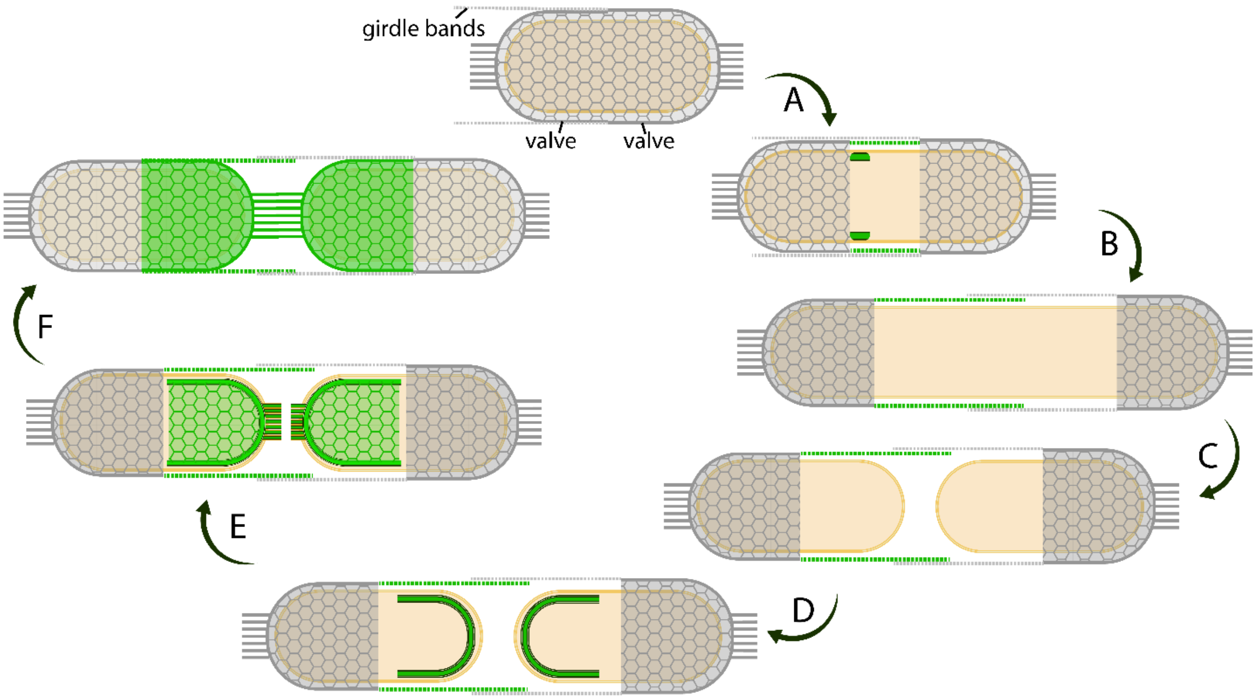

Figure S1. Diatom cell cycle as manifested in *Stephanopyxis turris*. New silica elements, formed during the current cell cycle are colored green. (**A**) Start of a cell cycle with a protoplast enclosed by the two parental valves, the valve rims are directly adjacent to each other. The older valve is on the right, and the younger valve on the left. The younger valve is covered by the girdle bands of the older valve. (**B**) Protoplast starts growing in preparation for the next cell division. As the silica valves are rigid and not elastic, growth is only possible in longitudinal direction by pushing apart the two valves. New girdle bands (green) are formed in individual SDVs and added to the rim of the younger valve. (**C**) After the protoplast has reached its final length, the cell undergoes cytokinesis, resulting in two daughter cells enclosed in the parental cell wall. (**D**) Each daughter cell inherits one half of the parental cell wall and starts forming a new valve, inside an SDV, shortly after cell division. (**E**) The new valves are completed with attachment of the linking extensions, after which they are exocytosed. (**F**) Both daughter cells are now ready to continue to the next cell cycle.

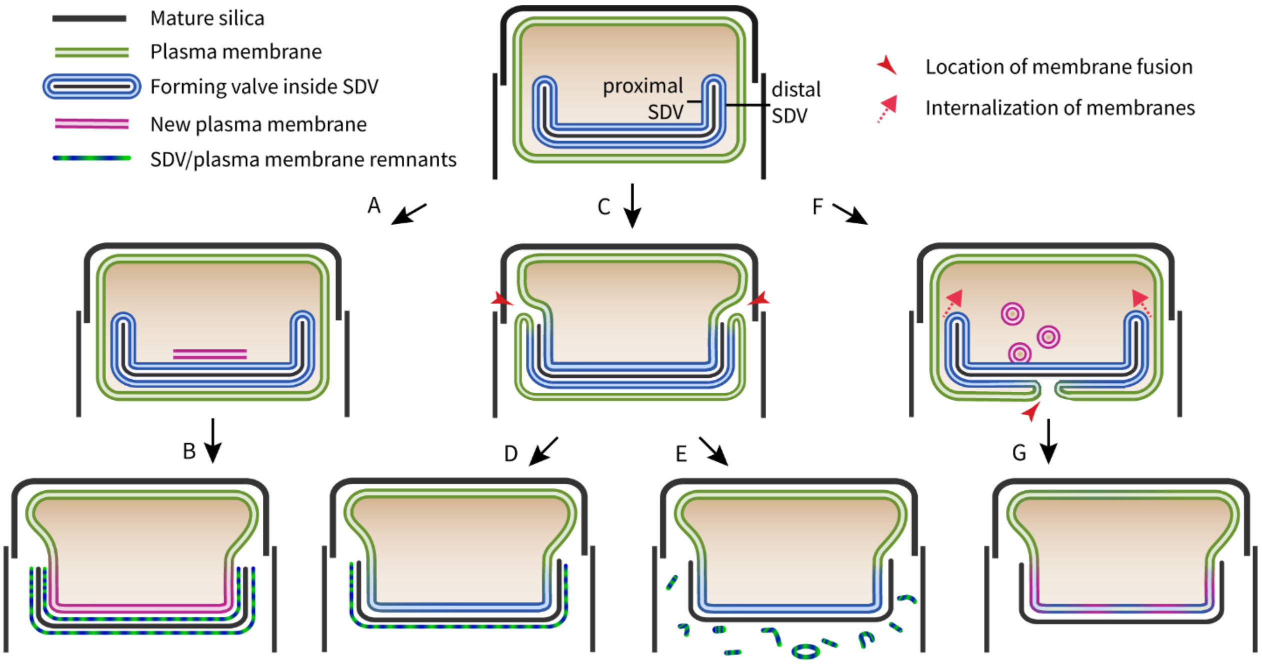

Figure S2. Hypotheses for valve exocytosis in diatoms. Early ultrastructural studies brought forward few models for silica cell wall exocytosis in diatoms that are reproduced here (14, 16–23). One scenario suggests that the newly formed valve is externalized through formation of a new plasma membrane underneath it (**A**). The SDV membrane and the original plasma membrane around the new valve remain in place, forming an organic layer around the silica (**B**). An alternative is that the valve is externalized as a result of localized fusion of the SDV and plasma membrane at the valve edge, such that the proximal part of the SDV membrane becomes the new plasma membrane (**C**). The distal part of the SDV membrane and the original plasma membrane were suggested to either shed off to the environment (**E**) or remain as an extracellular layer protecting the silica (**D**). The last model suggests localized membrane fusion at the apical part of the valve, followed by pulling of the distal SDV and original plasma membrane back into the cytoplasm at the valve edge (**F**). In this last model, which is in accordance with classical exocytosis, the composition of the proximal SDV membrane is altered for its new function as the plasma membrane (**G**). Our study supports model C+E as the scenario for valve exocytosis.

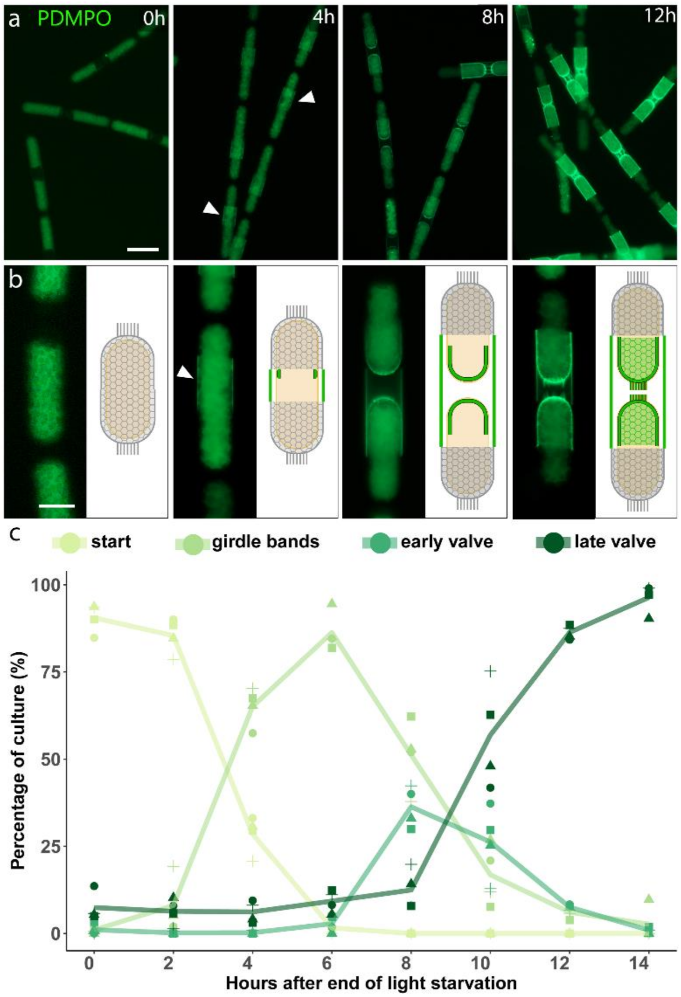

Figure S3. Light-induced synchronization of *S. turris* cell cycle. (**A**) Representative PDMPO fluorescence images from at least four independently synchronized cultures. Elapsed time since the end of light starvation and addition of PDMPO is indicated in the image. Shortly after the end of light starvation, none of the cells have fluorescently labelled silica, only PDMPO fluorescence from within the vacuoles is visible. Four hours later, most cells have started forming girdle bands, visible as a fluorescent ring (arrowheads) around the cell. After eight hours, the majority of the culture is forming new, fluorescent valves. After twelve hours, nearly all cells have completed the formation of a new valve. Scale bar: 50 µm. (**B**) Key stages of cell wall formation: 'start', cells without PDMPO-labelled silica; 'girdle bands', cells during cell elongation and girdle band formation; 'early valve', after cell division, valve formation has started but is prior to growth of linking extensions; 'late valve', cells at later stages of valve formation when polygons and

extensions are visible. Scale bar: 20 µm (**C**) Percentage of cells at key stages during synchronized growth. The lines show the mean of four independent experiments, indicated with symbols. For each time point at least 98 cells were counted. The highest percentage of cells that are forming valves is reached around nine hours.

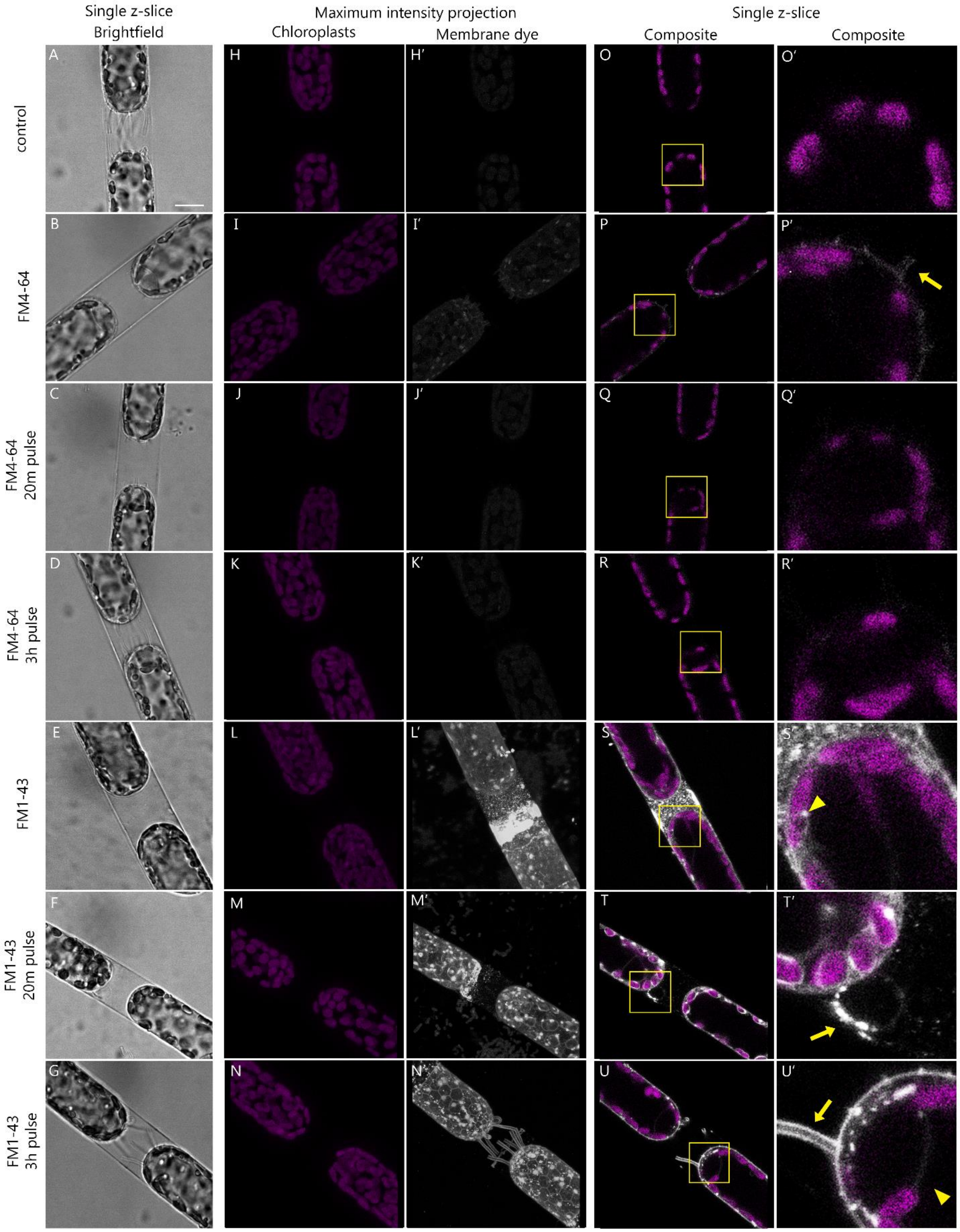
Single z-slice
Brightfield
Maximum intensity projection
Chloroplasts
Membrane dye
Single z-slice
Composite
Composite
control
FM4-64
FM4-64 20m pulse
FM4-64 3h pulse
FM1-43
FM1-43 20m pulse
FM1-43 3h pulse
A
B
C
D
E
F
G
H
H′
I
I′
J
J′
K
K′
L
L′
M
M′
N
N′
O
O′
P
P′
Q
Q′
R
R′
S
S′
T
T′
U
U′

Figure S4. Comparison of FM4-64 and FM1-43 labelling in *S. turris* cells. Representative micrographs of recently divided *S. turris* cells stained with FM4-64 or FM1-43. Each row shows the same cells, imaged using different fluorescence channels and using either 3D or 2D representations. (A, H-H' and O-O') control without any staining. (B, I-I' and P-P') continuously stained with FM4-64, as in Figure 2. The arrow indicates membrane staining. (C, J-J', and Q-Q') pulse-stained with FM4-64 for 20 minutes. (D, K-K' and R-R') pulse-stained with FM4-64 for 3 hours. In both pulse-stained experiments the FM4-64 labelling was washed off the cells. (E, L-L' and S-S') continuously stained with FM1-43. The signal intensity is higher relatively to FM4-64 (both acquisition settings were identical) as we did not optimize the minimal possible concentration as was done for the extensive FM4-64 experiments. (F, M-M' and T-T') pulse-stained with FM1-43 for 20 minutes. (G, N-N' and U-U') pulse-stained with FM4-64 for 3 hours. All FM1-43 experiments show internalization of the dye. Arrows: growing linking extensions surrounded by fluorescently stained membrane, arrowheads: internalized dyes staining intracellular compartments such as chloroplasts and nucleus. Staining experiments were repeated at least twice with consistency. Scale bar: 10 µm.

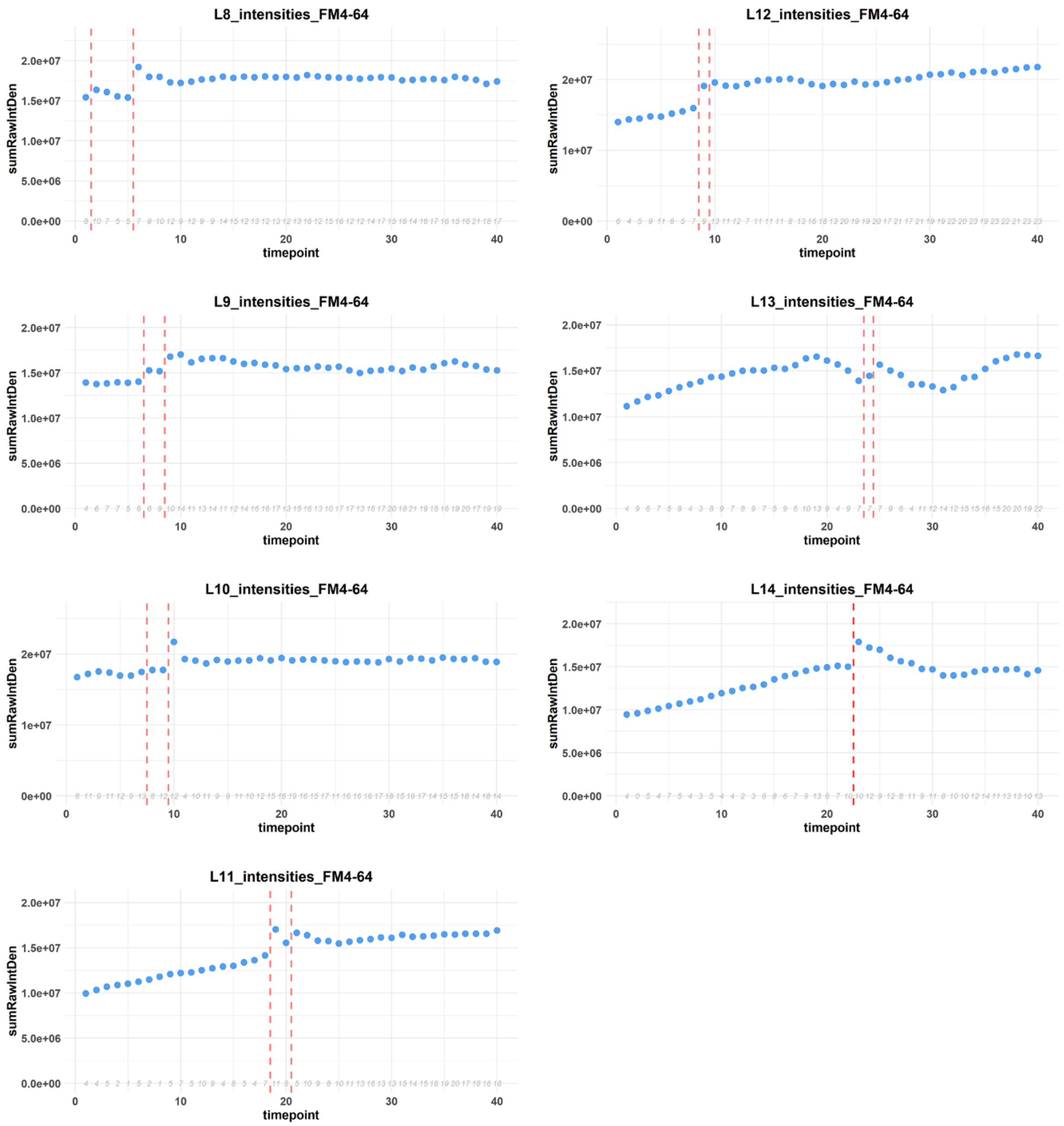

Figure S5. FM4-64 fluorescence intensity during live-cell imaging. Results from seven cells undergoing valve formation and exocytosis are shown. Total FM4-64 fluorescence intensity throughout time-lapses were measured using imageJ's 'raw integrated density (RawIntDen)'. This measure gives the sum of all pixel intensities in an image. Here we plotted for each time point the sum of RawIntDen of all slices in the z-stack. The red lines indicate where exocytosis happens, based on loosening of the hexagonal pattern. Thus, in 6 out the 7 cells (except for L13) a clear

fluorescence increase is associated with the disappearance of the polygonal pattern. In L14 only one of the two daughter cells remained in the field of view. Grey italic numbers indicate for each time point the number of slices that contain one or more oversaturated pixels, the Z-stacks contain 40 to 44 slices overall.

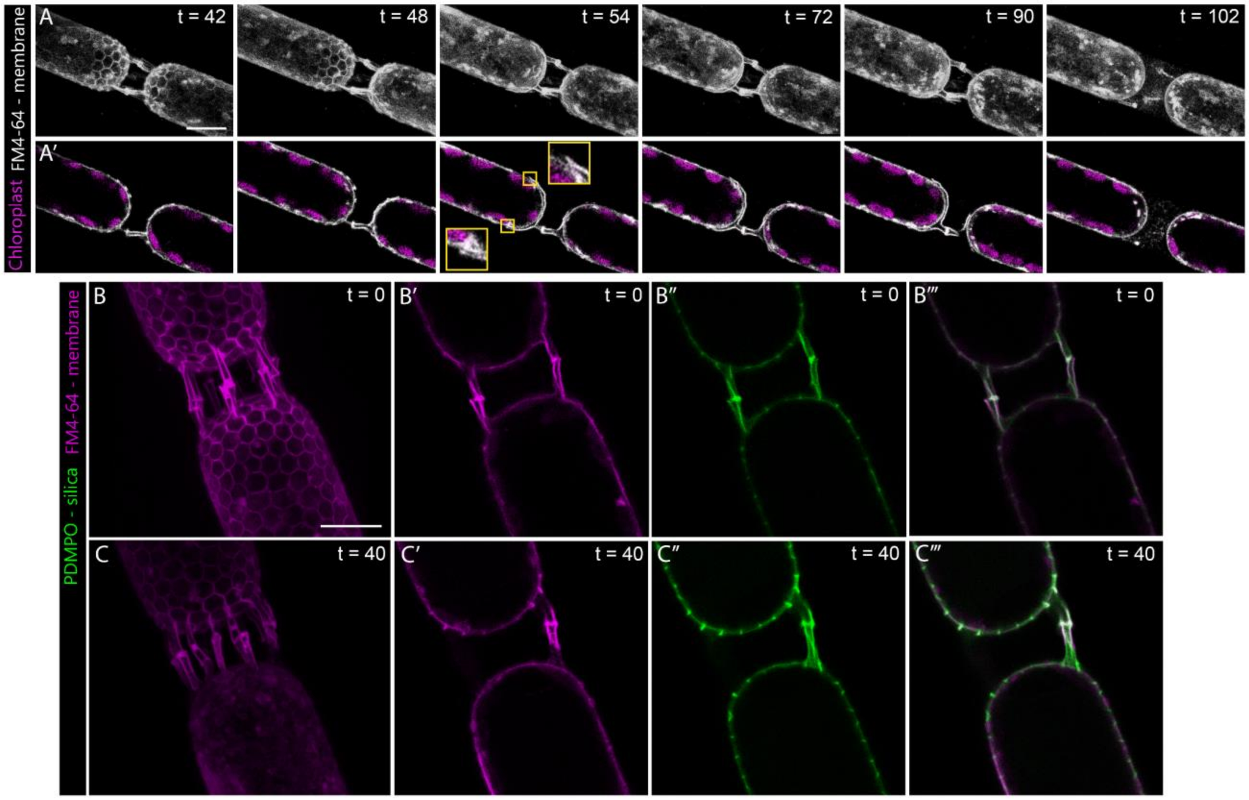

Figure S6. Distal membranes that undergo degradation are disconnected from the cellular membrane. Representative (**A**) maximum intensity projections and (**A'**) single z-slices of time-lapse confocal fluorescence images. Images show membrane staining (FM4-64) in white and chloroplasts autofluorescence in magenta. The elapsed time since the start of imaging (in minutes) is shown on the upper right corner. Exocytosis of the valve at the bottom right cell starts between t=42 and t=48. The valve on the top left is exocytosed between t=48 and t=54. In t=54 to t=90, distal membranes around the valve are not connected to the cellular membrane. At t=102 the remnants have largely disintegrated. 3D reconstructions (B-C) and single z-slices (B'-C''') of live-cell confocal fluorescence images. Images show membrane staining (FM4-64) in magenta and silica staining (PMDPO) in green. At time t=0 (B-B'''), both daughter cells have an intracellular valve, the membranes outline the polygonal silica structures. At the second imaging step (C-C''') the bottom valve has been exocytosed and the membrane stain no longer mirrors the silica stain. Scale bars: 10 µm.

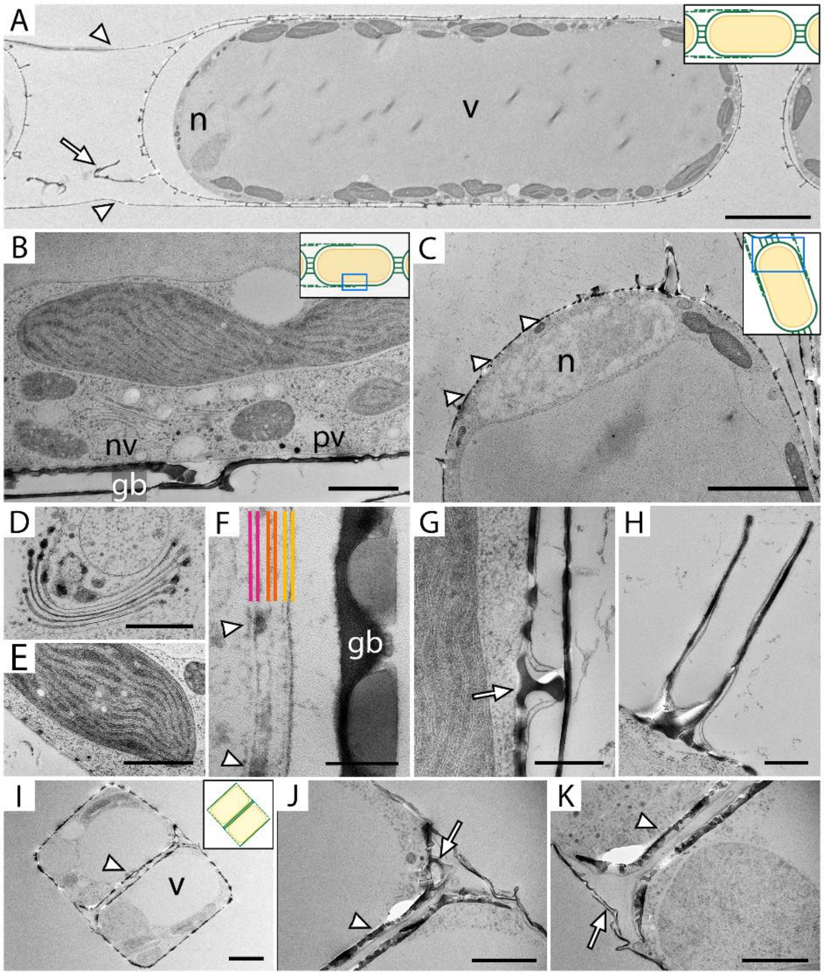

Figure S7. Ultrastructural details of diatoms revealed by TEM imaging. Insets indicate which part of the cell is displayed. (**A**) A cross section through an entire cell, the nucleus (n), and the large central vacuole (v) that is surrounded by darker chloroplasts are dominant. The silica cell wall (dark contrast) is clearly visible at the left side, where the protoplast is not directly adjacent to the cell wall due to slight plasmolysis. Arrowheads indicate girdle bands, and an arrow indicates a linking extension. Scale bar: 10 µm. (**B**) The meeting point of a new valve (nv), and parental valve (pv) with girdle bands (gb) attached to the latter. Scale bar: 1 µm. (**C**) Apex of a cell with the nucleus (n) located directly under the valve. Silica protrusions indicated with arrowheads are cross sections of the polygonal layer. Scale bar: 5 µm. (**D-H**) High magnification images of: (**D**) Golgi-body. Scale bar: 500 nm. (**E**) Chloroplast. Scale bar: 1 µm. (**F**) SDV with growing silica (arrowheads). Bilayers of the proximal SDV membrane (pink), distal SDV membrane (orange), and

plasma membrane (yellow) can be distinguished. Scale bar: 100 nm. (**G**) Fully formed polygon (arrow) still inside an SDV. Scale bar: 500 nm. (**H**) Linking extension during its formation in an SDV. Scale bar: 500 nm. (**I**) Cross section of two *T. pseudonana* daughter cells with intracellular valves (arrowheads). Scale bar: 1 µm. (**J-K**) Higher magnification of the cells in **I**, with arrow in **J** indicating a fultoportula in the forming valve and arrow in **K** indicating parental girdle bands. Freeze substitution was repeated at least three times with consistency. Scale bars: 500 nm.

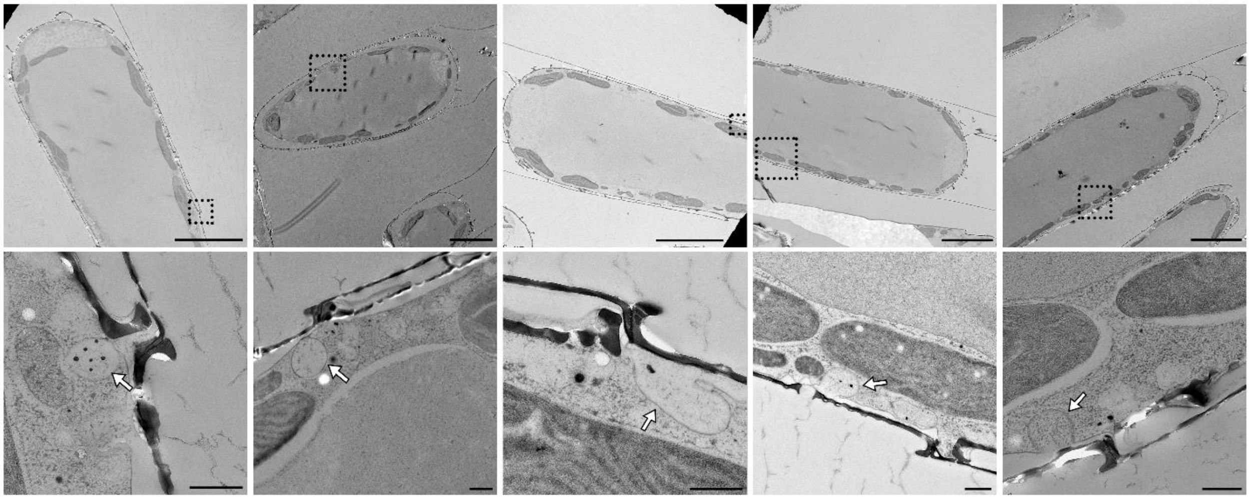

Figure S8. Instances of membrane invagination during valve exocytosis. Examples of cells that are undergoing valve exocytosis, i.e. have a mature valve that is exposed to the exterior but still surrounded by membrane (remnants). The bottom row shows magnified views of the boxed areas in the top row, arrows indicate membrane invaginations. Freeze substitution was repeated at least three times with consistency. Scale bars: 1 µm (top row) and 500 nm (bottom row).

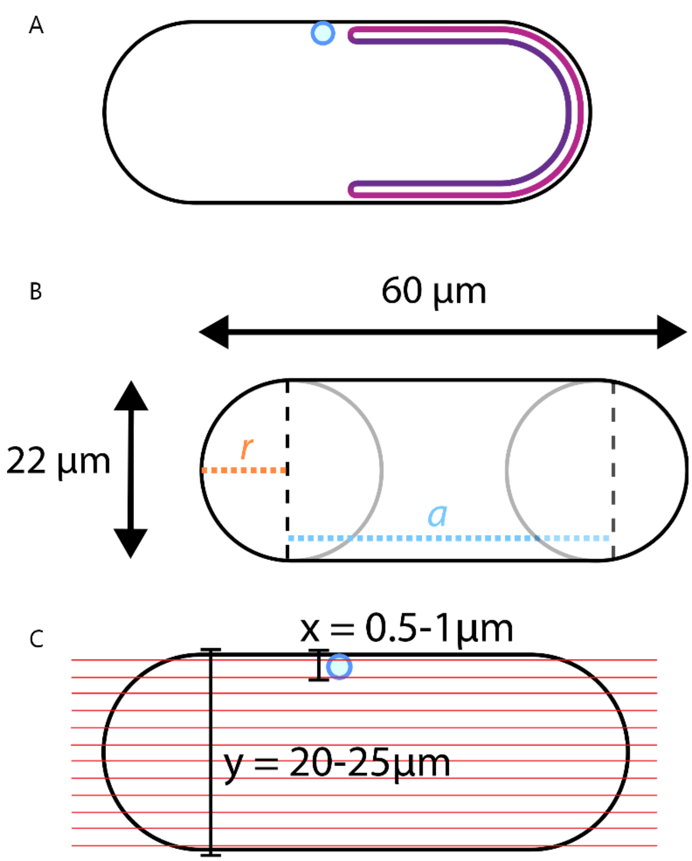

Figure S9. Geometrical dimensions of *S. turris* that were used in the statistical simulations. (A) Schematic of an *S. turris* cell with an SDV (pink and purple) and a putative endocytic vesicle (blue). Note that the length (in this 2D schematic) of the two sides of the SDV membrane is similar to the length of the entire plasma membrane. (B) Geometry of a capsule. (C) The probability of catching an endocytic vesicle of diameter x in a randomly taken cross section (red lines) of an *S. turris* cell with diameter y is x/y.

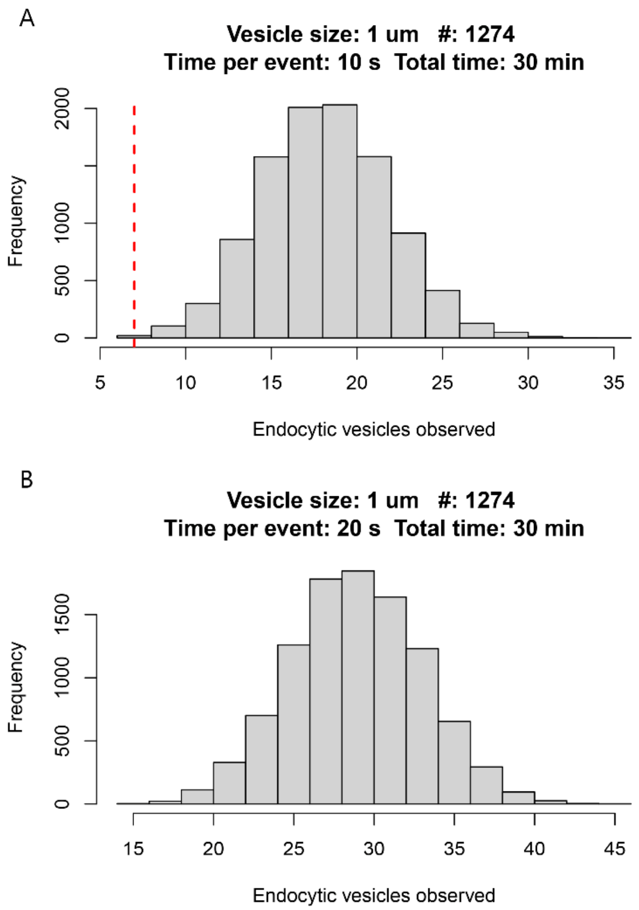

Figure S10. Histograms showing the distribution of 10,000 simulations according to the number of endocytic vesicles detected in them given 68 observations. In addition to the outcomes for a simulated scenario with invagination time of 10 seconds (A), we include a second scenario (B), in which the invagination time is 20 seconds. Red dashed line indicates our experimental result, we detected an endocytic vesicle in 7 out of 68 cells.
