# Supplementary material for: Exocytosis of the silicified cell wall of diatoms involves extensive membrane disintegration
Source: Nat Commun. 2023 Jan 30;14:480. doi: 10.1038/s41467-023-36112-z (PMC9886994; doi:10.1038/s41467-023-36112-z)
Supplement: Supplementary file 3 — Description for Additional Supplementary Files [file 41467_2023_36112_MOESM3_ESM.docx]

**Supplementary information**

**Exocytosis of the silicified cell wall of diatoms involves extensive membrane disintegration**

Diede de Haan, Lior Aram, Hadas Peled-Zehavi, Yoseph Addadi, Oz Ben-Joseph, Ron Rotkopf, Nadav Elad, Katya Rechav and Assaf Gal

**Supplementary movie captions**

**Movie S1.** Time-lapse imaging video of *S. turris* cell, stained with FM4-64, during valve formation and exocytosis.

**Movie S2.** Animation of cryo-ET dataset of *T. pseudonana* shown in Figure 4 A.

**Movie S3.** Animation of cryo-ET dataset of *T. pseudonana* shown in Figure 4 C.
